# Determinants of Non-calcified Low-Attenuation Coronary Plaque Burden in Patients Without Known Coronary Artery Disease: A Coronary CT Angiography Study

**DOI:** 10.3389/fcvm.2022.824470

**Published:** 2022-04-07

**Authors:** Hiroki Yamaura, Kenichiro Otsuka, Hirotoshi Ishikawa, Kuniyuki Shirasawa, Daiju Fukuda, Noriaki Kasayuki

**Affiliations:** ^1^Department of Cardiovascular Medicine, Kashibaseiki Hospital, Kashiba, Japan; ^2^Department of Cardiovascular Medicine, Osaka City University Graduate School of Medicine, Osaka, Japan

**Keywords:** chronic coronary syndrome (CCS), coronary CT angiography, high-risk plaque, coronary artery calcium score, epicardial adipose tissue, prognosis

## Abstract

**Background:**

Although epicardial adipose tissue (EAT) is associated with coronary artery disease (CAD), it is unclear whether EAT volume (EAV) can be used to diagnose high-risk coronary plaque burden associated with coronary events. This study aimed to investigate (1) the prognostic impact of low-attenuation non-calcified coronary plaque (LAP) burden on patient level analysis, and (2) the association of EAV with LAP volume in patients without known CAD undergoing coronary computed tomography angiography (CCTA).

**Materials and Methods:**

This retrospective study consisted of 376 patients (male, 57%; mean age, 65.2 ± 13 years) without known CAD undergoing CCTA. Percent LAP volume (%LAP, <30 HU) was calculated as the LAP volume divided by the vessel volume. EAT was defined as adipose tissue with a CT attenuation value ranging from −250 to −30 HU within the pericardial sac. The primary endpoint was a composite event of death, non-fatal myocardial infarction, and unstable angina and worsening symptoms requiring unplanned coronary revascularization >3 months after CCTA. The determinants of %LAP (Q4) were analyzed using a multivariable logistic regression model.

**Results:**

During the follow-up period (mean, 2.2 ± 0.9 years), the primary endpoint was observed in 17 patients (4.5%). The independent predictors of the primary endpoint were %LAP (Q4) (hazard ratio [HR], 3.05; 95% confidence interval [CI], 1.09–8.54; *p* = 0.033] in the Cox proportional hazard model adjusted by CAD-RADS category. Cox proportional hazard ratio analysis demonstrated that %LAP (Q4) was a predictor of the primary endpoint, independnet of CAD severity, Suita score, EAV, or CACS. The independent determinants of %LAP (Q4) were CACS ≥218.3 (*p* < 0.0001) and EAV ≥125.3 ml (*p* < 0.0001). The addition of EAV to CACS significantly improved the area under the curve (AUC) to identify %LAP (Q4) than CACS alone (AUC, EAV + CACS *vs*. CACS alone: 0.728 *vs*. 0.637; *p* = 0.013).

**Conclusions:**

CCTA-based assessment of EAV, CACS, and LAP could help improve personalized cardiac risk management by administering patient-suited therapy.

## Introduction

Coronary artery disease (CAD) is a progressive chronic disease that leads to acute coronary syndrome (ACS) often as the first manifestation (1). The assessment of myocardial ischemia is important for diagnosing patients who will benefit from coronary revascularization (2, 3). However, the ISCHEMIA trial (4) demonstrated that there was no significant differences in event-free survival between the invasive strategy (invasive coronary angiography and revascularization if necessary) plus optimized medical therapy (OMT) and conservative management with OMT alone. Therefore, it is important to identify high-risk patients treated with OMT who can develop subsequent coronary events.

Coronary plaque burden is a robust risk factor for plaque rupture, and it is the leading cause of ACS (3, 5, 6). There is a correlation between myocardial ischemia and coronary plaque burden; however, the disease progression of coronary atherosclerosis can be accelerated by the coexistence of distinct coronary risk factors such as metabolic activities (3, 7), which in turn account for the heterogeneity in the risk of developing unstable angina, myocardial infarction, and death among individuals. These findings suggest that there are potential mechanisms that accelerate the disease progression leading to ACS apart from myocardial ischemia (1, 3, 8). One of the mechanisms may involve an underlying high-risk coronary plaque composition in which earlier-stage plaques may have increased metabolic activities with a greater risk of plaque progression than advanced and more inert atherosclerotic lesions (3, 7, 9, 10). Coronary computed tomography angiography (CCTA) is a first-line, non-invasive diagnostic test to assess the presence, severity, and extent of CAD (11–13). It is mostly used in clinical practice because of its high negative predictive value for obstructive CAD. CCTA allows for fully quantitative assessment of coronary plaque burden, which has been shown to predict cardiovascular outcomes (7, 14, 15). A sub-analysis of the SCOT-HEART trial demonstrated that low-attenuation non-calcified coronary plaque (LAP) volume ≥4% was a robust predictor of the 5-year incidence of cardiac death or non-fatal myocardial infarction (16, 17). These observations indicate that quantifying high-risk coronary plaque burden is important to estimate the risk of future coronary events. Another mechanism that accelerates disease progression is inflammatory activity in the epicardial adipose tissue (EAT), a passive fat storage that functions as an endocrine organ (18). EAT volume (EAV) assessed using CCTA has been shown to play a pivotal role in coronary plaque progression (19–22). Even though EAV is associated with CAD extent, severity, and the presence of high-risk coronary plaque morphology (21, 23, 24), it is unclear whether EAV can be used to diagnose high-risk coronary plaque burden that progresses rapidly in patients without known CAD. In this CCTA study, we aimed to investigate (1) the prognostic impact of LAP burden on patient level analysis, as well as (2) the association of EAV with LAP volume in patients without known CAD.

## Materials and Methods

### Study Population

**Figure 1:**
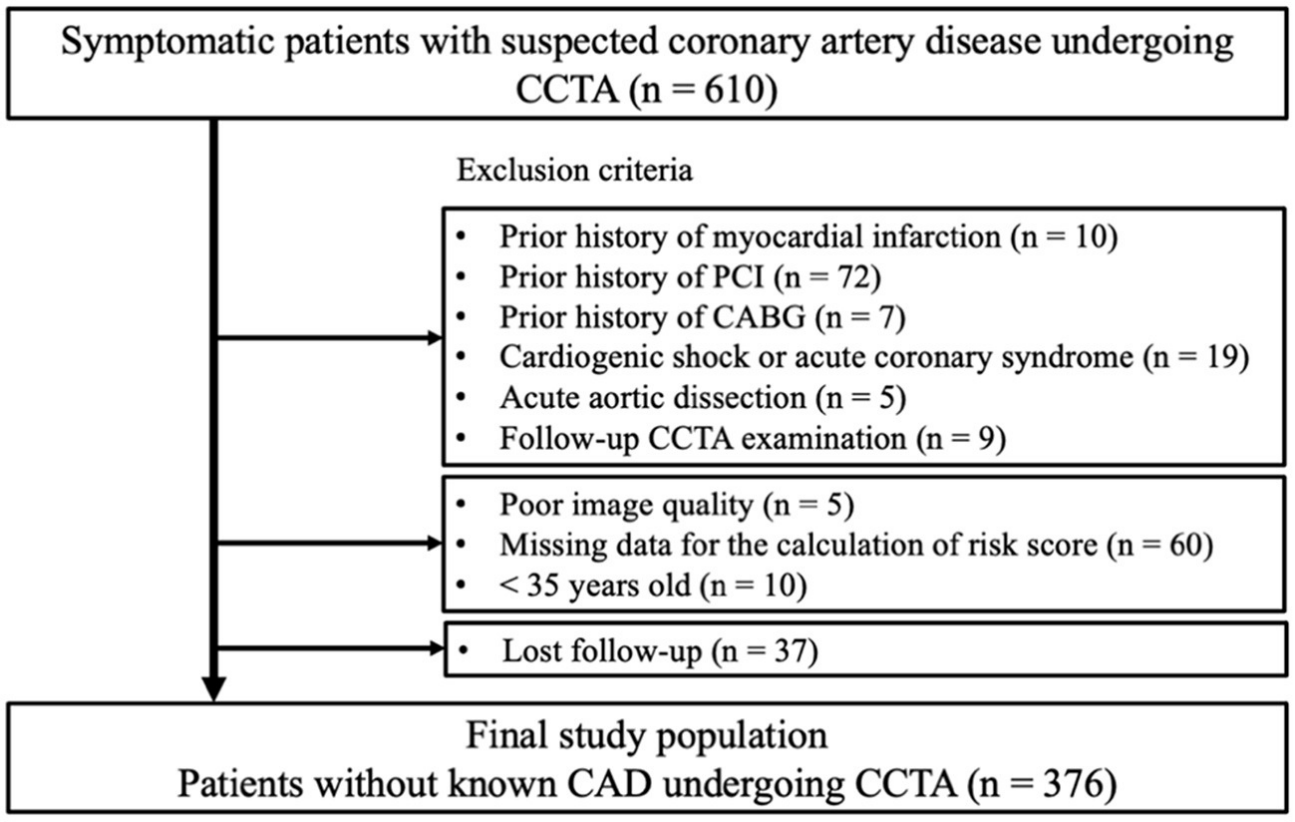
Flow chart for the study population. ACS, acute coronary syndrome; CAD, coronary artery disease; CCTA, coronary computed tomography angiography; CABG, coronary artery bypass graft; PCI, percutaneous coronary intervention.

This retrospective observational study protocol was approved by the ethics committee of Fujiikai Kashibaseiki Hospital (2021-E). The requirement of obtaining written informed consent from the participants was waived in accordance with the institutional requirements. The study was conducted in accordance with the Declaration of Helsinki. Figure 1 shows the flow chart of the study population. The study population consisted of symptomatic patients with suspected CAD (*n* = 610) who visited the Fujiikai Kashibaseiki Hospital between April 2017 and January 2020 for CCTA examination. Patients meeting the following criteria were excluded from this study: (1) patients with a prior history of myocardial infarction (*n* = 10), percutaneous coronary intervention (*n* = 72), or coronary artery bypass grafting (*n* = 7); (2) patients with cardiogenic shock or ACS (*n* = 19); (3) patients with acute aortic dissection (*n* = 5); (4) patients with second follow-up CCTA imaging (*n* = 9); (5) patients with a poor CCTA image quality (*n* = 5), missing data to calculate clinical risk score (*n* = 60), (6) those less than 35 years old who did not meet the criteria to calculate the clinical risk score (*n* = 10); and (7) patients who were lost to follow-up (*n* = 37). A total of 376 patients without known CAD were included in the analysis (Figure 1).

To estimate the 10-year probability of CAD, the Suita score was calculated and reported for each patient. The Suita score is a risk score that predicts the probability of developing of coronary heart disease in 10 years in the Japanese population (25). The parameters used to calculate the Suita score were age, sex, smoking status, blood pressure, low-density lipoprotein (LDL)-cholesterol, high-density lipoprotein (HDL)-cholesterol, and chronic kidney disease (CKD, estimated glomerular filtration ratio, eGFR <60 ml/min/1.73 mm^2^).

### CCTA Image Acquisition

All the CCTA examinations were performed using a 320-row multidetector CT and an ECG-triggered prospective gating method during a single breath-hold (Aquilion ONE/NATURE Edition, Cannon Medical Systems, Inc., Japan). Patients with a heart rate of >60 bpm were pretreated with an oral beta-blocker. Coronary artery calcium score (CACS) was assessed using the Agatston scoring method at a fixed thickness of 3 mm (26). Following CACS scanning, the Bolus Tracking method was used for the image acquisition, where the non-ionic contrast medium of 270 mgI/kg (ranging from 33 ml to 74 ml, Iopamidol, 370 mg iodine per ml iopamilon; Bracco, Milan, Italy) was administrated with a power injector at a rate of 2.3–4.9 ml per second through a 20-gauge needle. After injecting the contrast medium, saline was injected through the same venous access at the same injection rate. A region of interest (ROI) was set in the ascending aorta at the bronchial bifurcation level. When the CT value exceeded 150 Hounsfield units (HU), ECG-synchronized scans were performed within a single breath-hold. The scan parameters were a detector collimation of 0.5 × 320 mm, gantry rotation time of 350 ms, tube voltage of 120 kV, and tube current of 130–600 mA. The images were reconstructed using a Forward-projected Model-based Iterative Reconstruction SoluTion (FIRST) for coronary artery analysis.

### CCTA Image Analysis

Synapse Vincent software (Fujifilm Inc., Tokyo, Japan) was used to automatically generate three-dimensional volume-rendering images, straight and stretch curved planar reformation images, and cross-sectional multiplanar reconstruction (MPR) images. According to the Agatston scoring method, CACS was classified into five categories as follows: 0, 1–10, 11–100, 101–400 and >400. The diameter of the coronary artery stenosis was reported based on the SCCT guideline by two observers (K.O. and H.I.). According to the patient-level CAD-RADS classification (13), CAD was categorized into seven categories as follows: 0 (no plaque or no stenosis), 1 (1–24% stenosis), 2 (25–49% stenosis), 3 (50–69% stenosis), 4A (one or two vessels, 70–99% stenosis), 4B (left main artery >50% stenosis or three vessels ≥70% stenosis), and 5 (100%, total occlusion).

Regarding the vessel- and patient-level analysis of coronary plaque volume, we used the Synapse Vincent software (Fujifilm Inc.) to semiautomatically measure the lumen, vessel, and plaque volume of each major epicardial coronary artery. The luminal contour was automatically detected and manually corrected using cross-sectional MPR images. The vessel contour was automatically detected and manually corrected on the MPR images. The plaque components were categorized into either calcified plaque (CP, ≥150 HU), non-calcified plaque (NCP, <150 HU), or LAP <30 HU (17). The total coronary plaque burden and that for each plaque component was calculated using the following equation:


$$\begin{array}{l} \;Coronary\;plaque\;burden\;\left(\%\right)\\ =\;\frac{Vessel\;volume\;\left(ml\right)\;-\;Lumen\;volume\left(ml\right)}{Vessel\;volume\;\left(ml\right)}\;\times \;100 \end{array}$$

**Figure 2:**
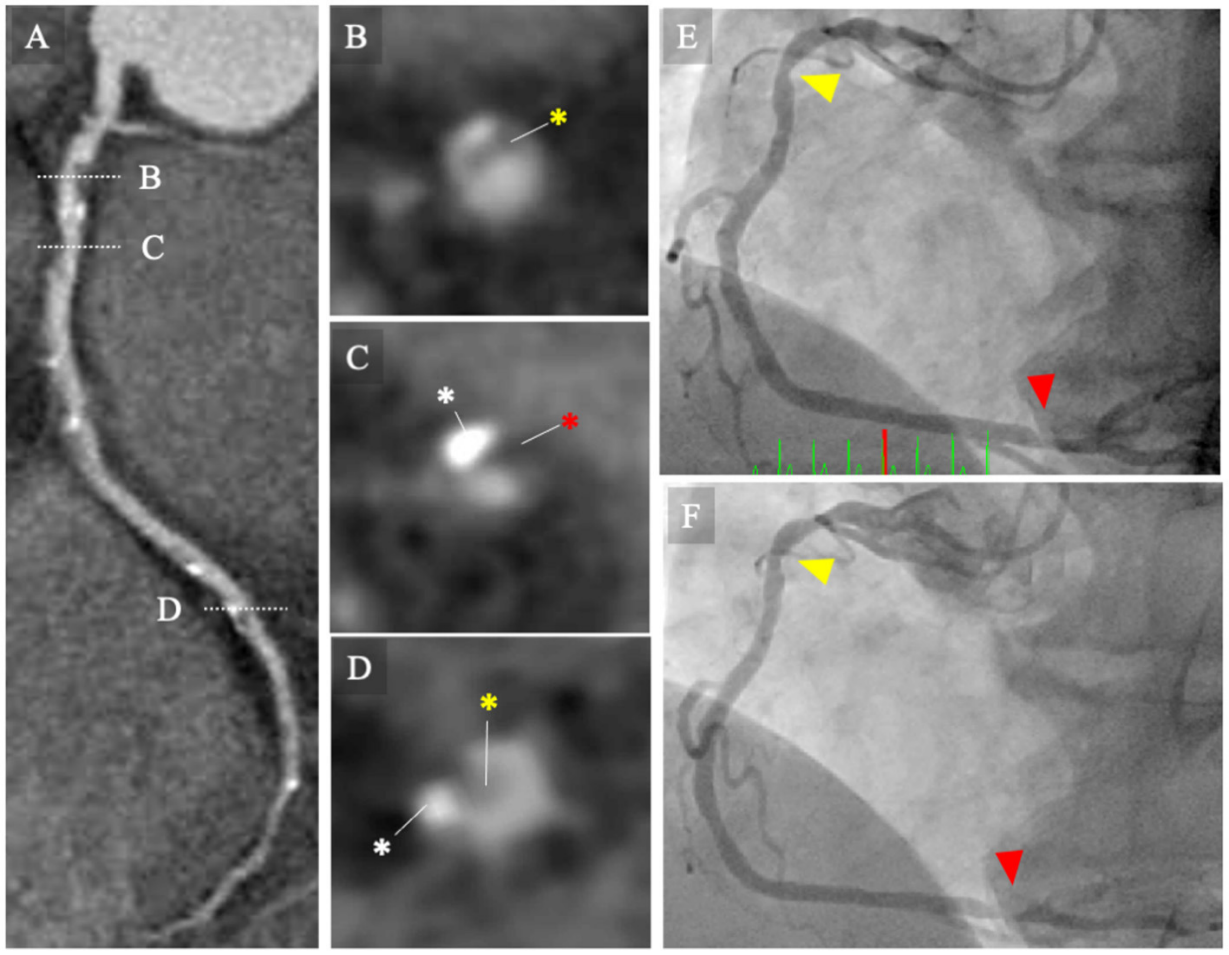
CCTA images and invasive coronary angiography. **(A–D)** CCTA images of a patient with non-obstructive CAD and increased low-attenuation plaque (Q4) who developed to unstable angina requiring urgent coronary revascularization. The MPR image of the baseline CCTA image for right coronary artery (RCA) showing intermediate stenosis severity with low-attenuation coronary plaque (Q4). **(B–D)** Cross-sectional images of coronary lesions (white broken bars). **(B)** Mild stenosis with non-calcified plaque (yellow asterisk). **(C)** Intermediate stenosis with calcified plaque (white asterisk) and low-attenuation plaque (red asterisk). **(D)** Intermediate stenosis with calcified and non-calcified plaques. **(E)** Invasive coronary angiography performed at 12 days following the baseline CCTA examination. Invasive coronary angiography (ICA) image shows intermediate stenosis of the proximal (yellow arrowhead) and distal portions of the RCA (red arrowhead). The patient was managed with conservative strategy, including statins. **(F)** The patient presented with unstable angina and underwent emergency ICA at 1.5 years following the baseline CCTA examination. The ICA image revealed progression of the coronary lesions (red arrowheads). CCTA, coronary computed tomography angiography; ICA, invasive coronary angiography; MPR, multi-planar reconstruction; RCA, right coronary artery.

In the patient-level analysis, each plaque volume for 3-vessel coronary arteries was categorized into quartiles. Figure 2 illustrates the CCTA images (Figures 2A–D) of a patient with non-obstructive CAD and an increased LAP (Q4) who developed unstable angina requiring urgent coronary revascularization.

EAV was measured from contrast-enhanced CT images using the Synapse Vincent software (Fujifilm Inc.) as reported previously (27). To measure the EAV, several equidistant axial planes were extracted according to the size of each heart. The upper slice limit was at the bifurcation of the pulmonary artery trunk, and the lower slice limit was at the last slice that contained any structure of the heart. In each plane, the software automatically detected a smooth, closed pericardial contour as a region of interest, where the software automatically identifies adipose tissue as having CT attenuation values ranging from −250 to −30 HU within the pericardial sac (27). Finally, the EAV was calculated as the sum of the EAT areas.

### Endpoints

Clinical follow-up was performed through interviews with the patients at each hospital visit. Interviews through telephone and mail were used to obtain the follow-up data. The primary endpoint was defined as a composite event of cardiovascular or non-cardiovascular death, non-fatal myocardial infarction, and unstable angina requiring coronary revascularization more than 90 days after CCTA examination, as well as worsening symptoms requiring unplanned coronary revascularization more than 90 days after CCTA examination. Myocardial infarction was defined as a typical persistent chest pain with elevation of cardiac enzymes (elevation of cardiac troponin I above the reference level), regardless of ST-segment elevation myocardial infarction or non-ST-segment elevation myocardial infarction. Unstable angina was defined as new-onset angina, exacerbated angina that is symptomatic with light exertion, or angina that appears at rest, without elevated cardiac-deviated enzymes.

### Statistical Analysis

Statistical analysis was performed using EZR (Saitama Medical Center, Jichi Medical University, Saitama, Japan), which is a modified version of the R commander. Categorical variables were reported as counts (percentage), and continuous variables as means (standard deviation, SD) or medians (interquartile range, IQR). The variables were compared using the chi-square test for categorical variables. One-way analysis of variance or the Kruskal-Wallis test was used to compare the %LAP quartiles for continuous variables. A Cox proportional hazard ratio analysis (forced entry method) was used to identify the predictors of the primary endpoint. The multivariable model using Cox hazard proportional ratio analysis included 4 models; LAP (Q4) was adjusted by CAD-RADS ≥3 for model 1, Suita score for model 2, EAV for model 3, and CACS >100 for model 4. Using the Kaplan-Meier curve analysis, a time-to-event analysis for patients with %LAP Q4 and those with %LAP Q1–3 was performed. The determinants of %LAP (Q4) were analyzed using logistic regression analysis. The model was adjusted for advanced age, body mass index, Suita score, and obstructive CAD. Suita scores of <56 and ≥56 points were classified as low risk (*n* = 274; <9 % for 10-year risk) or intermediate/high risk (*n* = 102; ≥9 % for 10-year risk), respectively. Spearman's correlation coefficient was used to analyze the association of %LAP with CACS and EAV. Receiver operating curve (ROC) analyses were performed to determine the best cutoff value of EAV and CACS in identifying patients with increased %LAP (Q4) and the areas under the curve (AUCs) as well as the sensitivities, specificities, and positive and negative predictive values of the diagnostic test. To test the hypothesis that EAV had additional diagnostic value than CACS, a multivariate ROC analysis adjusting for EAV and CACS was performed and compared with that of CACS alone. To report the reproducibility of coronary plaque burden, we performed an intraclass correlation (ICC) analysis for intra- and interobservers in 30 randomly selected patients. A *p*-value of < 0.05 was considered as statistically significant.

## Results

### Baseline Characteristics and CCTA Findings

All of the 376 patients who underwent CCTA examination had images of suitable quality as well as available clinical data for analysis. Table 1 shows the baseline characteristics of the patients. The patients had a mean age of 65.2 (13.1) years, and 56.6% (*n* = 213) were male with a range of cardiovascular risk factors. The mean Suita score was 48.4 (10.8) points, and 27.1% (*n* = 102) of the patients had Suita scores ≥56 (Table 1). Table 1 shows that 5.0% (*n* = 19) and 25.5% (*n* = 96) of the patients were treated with aspirin and statins at study enrollment, respectively. Compared to patients without the primary endpoint, those with the primary endpoint tended to have greater prevalence of diabetes and were older (*p* = 0.011), as well as had a higher Suita scores (*p* = 0.003). There was no significant difference in serum CRP (*p* = 0.502) and eGFR (*p* = 0.161) levels between patients with and without the primary endpoint. Table 2 shows patient-level CCTA findings at baseline and a comparison between patients with and without the primary endpoint. The median CACS was 17 (0–166). Accoridng to CAD-RADS category, CAD-RADS 1 (absence of CAD), CAD-RADS 1–2 (minimal-mild CAD), and CAD-RADS ≥3 (severe stenosis and total occlusion) were found in 20.2% (*n* = 76), 39.6% (*n* = 149), and 40.2% (*n* = 151) of the patients, respetively. The mean %LAP for all the patients was 1.35 (0.98) % and the mean EAV was 124.0 (52.0) ml. As expected, patients with the primary endpoint had higher prevalence of CAD-RADS ≥3, and a greater plaque volume for calcified plaque and low-attenuation non-calcified plaque (all *p* < 0.05), but there were no statistically significant differences in EAV between the two groups (*p* = 0.40).

**Table 1:** Baseline patient characteristics.

	**Overall** **(*n* = 376)**	**Primary endpoint (+) (*n* = 15)**	**Primary endpoint (-) (*n* = 361)**	***p*-value**
Age, years	65.2 (13.1)	73.7 (9.8)	64.9 (13.1)	0.011
Male	213 (56.6 %)	10 (66.7%)	203 (56.2%)	0.424
Body mass index, kg/m^2^	24.0 (4.1)	22.8 (3.0)	24.0 (4.1)	0.236
Smoking	58 (15.4 %)	2 (13.3%)	56 (15.5%)	0.819
Hypertension	269 (71.5 %)	10 (66.7%)	259 (71.7%)	0.669
Diabetes mellitus	84 (22.3 %)	6 (40.0%)	78 (21.6%)	0.094
Dyslipidemia	275 (73.1 %)	12 (80.0%)	263 (72.9%)	0.541
Atrial fibrillation	45 (12.0 %)	1 (6.7%)	44 (12.2%)	0.519
CKD	101 (26.9 %)	5 (33.3%)	96 (26.6%)	0.564
eGFR, ml/min/1.73 mm^2^	67.9 (13.8)	63.0 (21.6)	68.2 (13.4)	0.161
LDL-Cholesterol, mg/dL	125 (35.1)	133.3 (25.2)	124.8 (35.4)	0.355
HDL-Cholesterol, mg/dL	63.7 (18.6)	61.4 (17.8)	63.8 (18.6)	0.615
Triglyceride, mg/dL	156 (216)	153.2 (87.4)	157.1 (219.8)	0.946
CRP, mg/dL	0.33 (0.84)	0.18 (0.29)	0.33 (0.86)	0.502
Hemoglobin A1c, %	6.0 (1.1)	6.1 (0.6)	6.0 (1.1)	0.897
**Medication**				
Aspirin	19 (5.0 %)	0 (0%)	19 (5.3%)	0.362
Beta blockers	22 (5.9 %)	0 (0%)	22 (6.1%)	0.324
RAS-inhibitors	86 (22.9 %)	4 (26.7%)	82 (22.7%)	0.581
Calcium channel blockers	102 (27.1 %)	5 (33.3%)	97 (26.9%)	0.182
Statins	96 (25.5 %)	5 (33.3%)	91 (25.2%)	0.479
Suita score	48.4 (10.8)	56.4 (9.8)	48.1 (10.7)	0.003

*Variables were expressed as n (%) or mean (SD). eGFR, estimated glomerular filtration rate; CKD, chronic kidney disease; CRP, C-reactive protein; HDL, high-dense lipoprotein; LDL, low-dense lipoprotein; RAS, renin-angiotensin system*.

**Table 2:** Baseline patient-level CCTA findings.

	**Overall** **(*n* = 376)**	**Primary endpoint (+) (*n* = 15)**	**Primary endpoint (-) (*n* = 361)**	***p*-value**
**CAD-RADS classification**				
0	76 (20.2 %)	1 (6.7%)	75 (20.8%)	0.182
1	110 (29.3%)	1 (6.7%)	109 (30.2 %)	0.049
2	39 (10.3%)	3 (20.0%)	36 (10.0%)	0.212
3	74 (19.7%)	1 (6.7%)	73 (20.2%)	0.196
4A	44 (11.7%)	3 (20.0%)	41 (11.4%)	0.308
4B	21 (5.6%)	3 (20.0%)	18 (5.0%)	0.0131
5	12 (3.2%)	3 (20.0%)	9 (2.5%)	<0.001
≥3	151 (40.2 %)	10 (66.7%)	141 (39.1%)	0.033
**Location of obstructive CAD**				
LMCA	8 (2.1 %)	1 (6.7%)	7 (1.9%)	
LAD	105 (27.9 %)	9 (60.0%)	96 (26.6%)	
LCX	57 (15.2 %)	5 (33.3%)	52 (14.4%)	
RCA	59 (15.7 %)	6 (40.0%)	53 (14.7%)	
%NCP volume, %	21.7 (6.52)	23.4 (6.53)	21.7 (6.52)	0.302
%CP volume, %	1.03 (2.82)	6.22 (9.17)	0.82 (1.97)	<0.001
%LAP volume, %	1.35 (0.98)	1.92 (1.28)	1.33 (0.96)	0.023
CACS, Agatston unit	17 (0–166)	67 (0–390)	4.8 (0–108)	<0.001
EAV, ml	124.0 (52.0)	134.9 (51.0)	123.5 (51.2)	0.4
Abdominal visceral adipose tissue area, cm^2^	101.3 (57.4)	110.4 (66.0)	100.9 (57.1)	0.53

*Variables were expressed as n (%), mean (SD) or median (interquartile range, IQR). CACS = coronary artery calcium score, CAD, coronary artery disease; CCTA, coronary computed tomography angiography; EAV, epicardia adipose tissue volume; LAD, left anterior ascending artery; LCX, left circumflex artery; LMCA, left main coronary artery; RCA, right coronary artery; NCP, non-calcified plaque; CP, calcified plaque; LAP, low attenuation non-calcified plaque*.

Excellent reproducibility was observed in %LAP volume (intraobserver ICC, 0.939; 95% CI, 0.887–0.96; interobserver ICC, 0.953; 95% CI, 0.900–0.978), %NCP volume (intraobserver ICC, 0.981; 95% CI, 0.966–0.991; interobserver ICC, 0.977; 95% CI, 0.952–0.989), and %CP volume (intraobserver ICC, 0.989; 95% CI, 0.979–0.994; interobserver ICC, 0.984; 95% CI, 0.966–0.992).

### Predictors of the Primary Endpoint

During the mean follow-up period of 2.2 ± 0.9 years, the primary endpoint was observed in 15 patients (4.0%), including death (*n* = 2), ACS (*n* = 6), and unplanned coronary revascularization more than 90 days after CCTA examination (*n* = 7). Table 3 shows the comparisons of the Suita score, CCTA findings, and the rates for the primary endpoints among the four groups stratified by %LAP (Q1-Q4). Further, %LAP in each quartile was 0.07–0.72% for Q1 (25 percentile), 0.72–1.06% for Q2 (25–50 percentile), 1.07%−1.66% for Q3 (50–75 percentile), and 1.67–7.09% for Q4 (75–100 percentile). Of the patients who developed the primary endpoints, 65% (*n* = 11) arose from %LAP (Q4), which was more frequent than the remaining groups of %LAP (Q1–Q3). Compared to patients with the %LAP (Q1–Q3), those with %LAP (Q4) had a greater Suita score, CACS, EAV, and frequent obstructive CAD (all *p* < 0.05) (Table 3).

**Table 3:** Clinical risk score, CCTA findings, and event rates according to quartile of %LAP.

	**%LAP Q1 *n* = 94**	**%LAP Q2 *n* = 94**	**%LAP Q3 *n* = 94**	**%LAP Q4 *n* = 94**	***p*-value**
%LAP, %	0.52 (0.15)	0.88 (0.10)	1.33 (0.16)	2.67 (1.08)	<0.001
Suita score	45.2 (11.3)	48.6 (9.6)	49.6 (11.2)	50.3 (10.3)	0.006
CACS	0 (0–64)	15 (0–93)	24 (0–169)	67 (0–390)	<0.001
EAV, mL	102.1 (44.0)	112.6 (43.7)	132.4 (51.0)	149.0 (53.0)	<0.001
Obstructive CAD	28 (29.8)	36 (38.3)	39 (41.5)	48 (51.1)	0.029
Number of patients with primary endpoints	2 (2.1%)	1 (1.1%)	4 (4.3%)	8 (8.5%)	0.046

*Variables were expressed as n (%), mean (SD) or median (interquartile range, IQR)*.

*Abbreviations as in Table 2*.

**Figure 3:**
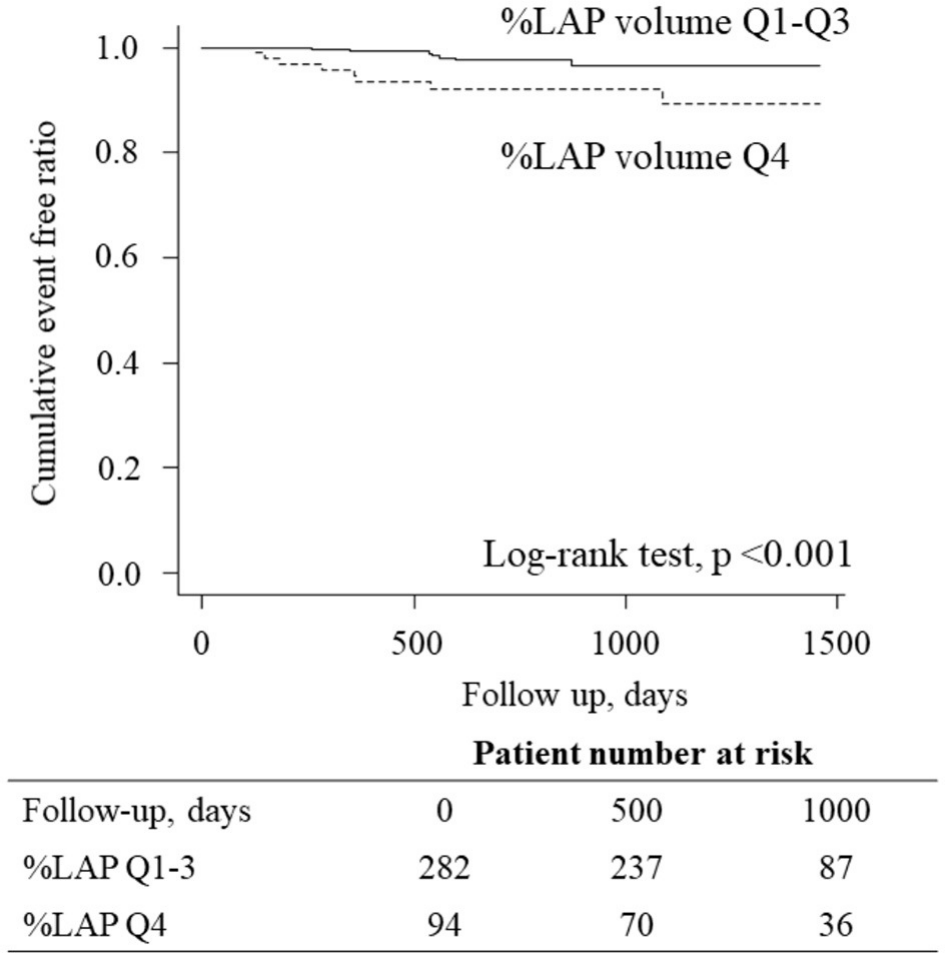
Kaplan-Meier curves analysis according to the LAP burden. Kaplan-Meier curve analysis illustrated that patients having %LAP (Q4) had a worse prognosis than those with Q1–Q3 (*p* < 0.001, log-rank test).

In the Cox proportional hazard model adjusted by CAD-RADS ≥3 (model 1), %LAP (Q4) was independent predictors of the primary endpoint (hazard ratio [HR], 3.05; 95% confidence interval [CI], 1.09–8.54; ***p*** = 0.033) (Table 4). Similarly, %LAP (Q4) remained as the predictor of the primary endpoint in model 2 adjusted by Suita score (LAP Q4; HR, 3.41; 95% CI, 1.23–9.45; *p* = 0.018), in model 3 adjusted by EAV (LAP Q4; HR, 3.15; 95% CI, 1.06–9.32; *p* = 0.038), and in model 4 adjusted by CACS ≥100 (LAP Q4; HR, 3.52; 95% CI, 1.28–9.71; *p* = 0.015) (Table 4). Kaplan-Meier curve analysis illustrated that patients with %LAP (Q4) had a worse prognosis than those with Q1–Q3 for both endpoints (both log rank *p* < 0.001) (Figure 3).

**Table 4:** Multivariable Cox hazard model for the prediction of the primary and secondary endpoints during follow-up.

	**Predictors of the primary endpoint**			
	**Hazard ratio**	**95% CI lower**	**95% CI upper**	***p*-value**
**Model 1**				
%LAP Q4	3.05	1.09	8.54	0.033
CAD-RADS ≥3	2.77	0.93	8.22	0.066
**Model 2**				
%LAP Q4	3.41	1.23	9.45	0.018
Suita score ≥56	2.37	0.86	6.55	0.096
**Model 3**				
%LAP Q4	3.15	1.06	9.32	0.038
EAT volume	1.45	0.48	4.37	0.511
**Model 4**				
%LAP Q4	3.52	1.28	9.71	0.015
CACS >100	1.45	0.41	5.65	0565

*Abbreviations as in Tables 1, 2. A Cox proportional hazard model (forced entry method) was carried out to identify the predictors of the primary endpoint. The predictors included in the model were %LAP (Q4), CACS >300, EAV, obstructive CAD, and Suita score ≥56*.

### Predictors of Patients With %LAP (Q4)

**Figure 4:**
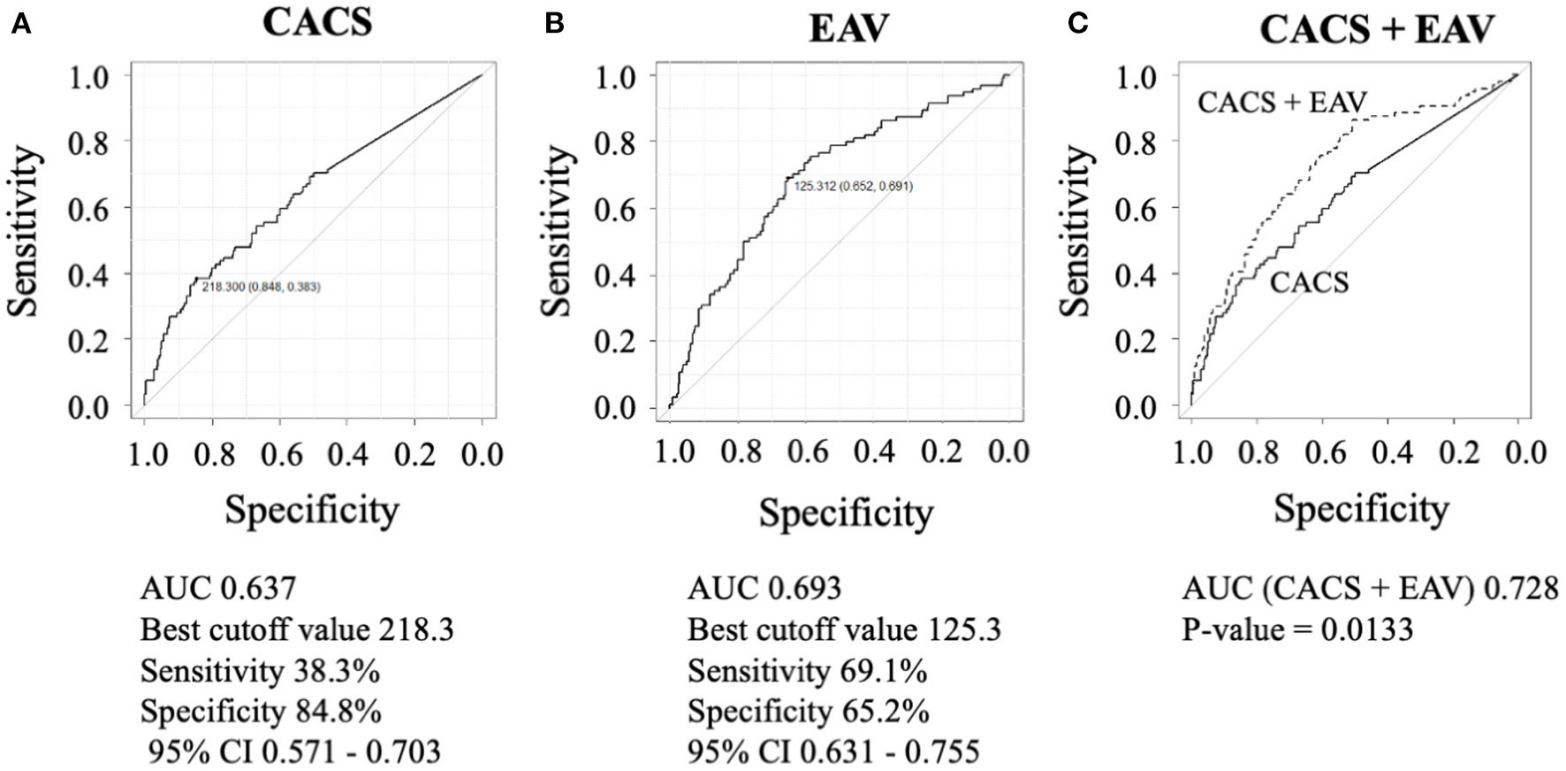
To diagnose patients with %LAP (Q4), the ROC analysis demonstrated that the best cut-off value for CACS was 218.3 Agatston units **(A)**, and that for EAV was 125.3 ml **(B)**. **(C)** Addition of EAV on CACS significantly improved the AUC that is used to identify %LAP (Q4) than CACS alone (C, EAV + CACS versus CACS alone, 0.728 versus 0.637; p = 0.013).

We observed a weak but statistically significant positive correlation between CACS and %LAP (ρ = 0.238, *p* < 0.001) and between EAV and %LAP (ρ = 0.386, *p* < 0.001). To diagnose patients with %LAP (Q4), the ROC analysis demonstrated that the best cut-off value for CACS was 218.3 Agatston units (Figure 4A, AUC = 0.637, sensitivity of 38.3%, and specificity of 84.8%), and that for EAV was 125.3 ml (Figure 4B, AUC = 0.693, sensitivity of 69.1%, and specificity of 65.2%). In a logistic regression analysis after adjusting for age, body mass index, Suita score, and CAD-RADS category, the independent determinants of %LAP (Q4) were CACS ≥218.3 and EAV ≥125.3 ml (both *p* < 0.001, Table 5). For the identification of patients with %LAP (Q4), the addition of EAV to CACS significantly improved the AUC (Figure 4C, AUC, 0.728, *p* = 0.013) compared to that of CACS alone (Figure 4A, AUC, 0.637).

**Table 5:** Multivariable logistic regression analysis to predict %LAP (Q4).

	**Odds ratio**	**95% CI lower**	**95% CI upper**	***p*-value**
Age >70 years	0.918	0.515	1.64	0.772
Body mass index, kg/m^2^	1.06	0.990	1.14	0.093
Suita score ≥56	0.939	0.516	1.71	0.838
CACS ≥218.3 Agatston unit	3.38	1.84	6.20	<0.001
EAV ≥125.3 ml	3.14	1.73	5.69	<0.001
Obstructive CAD	1.29	0.751	2.23	0.353

*Abbreviations as in Table 2. Suita score ≥56: probability of coronary heart disease in 10 years ≥9%*.

## Discussion

Our results show that in patients without overt known CAD, 3-vessel LAP volume is a significant predictor of the primary and secondary endpoints independent of CAD-RADS ≥3 (severe stenosis and total occlusion). The major findings of the present study are as follows: (1) Coronary high-risk plaque burden, assessed by LAP volume on CCTA, was a prognostic factor for mortality and coronary events; (2) EAV was independently associated with increased LAP volume; and (3) the combination of CACS and EAV provides a non-invasive method of identifying patients with increased LAP volume that can lead to coronary events. These findings suggest that the link between high-risk coronary plaque burden, death, and acute coronary events is potentially mediated by increased EAT volume.

Our understanding of the mechanisms of ACS largely stems from histopathological studies followed by investigations using imaging modalities (5, 28–32). The identification of patients at risk of developing ACS who will benefit from more intensive therapy is the focus of current clinical practice (11, 12, 31). Although the concept of rupture-prone “vulnerable plaque” was a driving motivation of the identification of patients at risk of ACS (5, 29), there are debates regarding how to diagnose patients who are most likely to develop coronary atherosclerotic disease burden leading to ACS (3).

Calcification plays a vital role in the natural history of coronary atherosclerosis and plaque rupture. Microcalcification (≥0.5 μm, and typically <15 μm) derived from dying macrophages and smooth muscle cells potentially causes stress-induced fibrous cap rupture (6, 33, 34). Several clinical studies using imaging modalities have demonstrated that spotty calcification is frequently found in the culprit lesion as compared to that of stable angina (28). It has been reported that high-density calcification on CCTA is a marker of plaque stability (10). Mortensen et al. recently demonstrated that CACS provides prognostic information regardless of the presence or absence of obstructive CAD (7), indicating the importance of assessing coronary plaque burden. These findings indicate that coronary lesions with mild-to-intermediate calcification exhibit a higher incidence of ACS (33). In lin with these findings, our results indicate that CACS ≥218.3 was independently associated with a greater high-risk plaque burden, as assessed by %LAP.

In recent years, atherosclerosis has been established as a chronic inflammatory disease (8). Adipose tissue is a passive fat storage and can function as an endocrine organ, releasing adipokines in response to extracellular stimuli or alterations in metabolic status (18). There is a link between local inflammation in EAT and lipid-rich coronary plaques and necrotic core, likely mediated by an increased density of vasa vasorum (35). Previous studies have reported an increased density of neovascularization from the adventitia into ruptured plaques (6, 36, 37), where increased expression of matrix metalloproteinases-2 and−9 by macrophages was observed in inflamed adventitia (38). As a result, more attention has been paid to assessing the inflammatory status of the pericoronary artery using non-invasive imaging modalities (39).

EAV has been independently associated with the extent and severity of obstructive CAD (21), hemodynamically significant stenosis (40), and the presence of high-risk coronary plaque detected on CCTA (24). Pericoronary fat volume has previously been reported to be significantly correlated with the presence of both calcified plaques and non-calcified plaques, indicating that perivascular fat depots play a pivotal role in the local process of atherosclerotic disease progression (19). Furthermore, Schlett et al. demonstrated that EAT volume is associated with high-risk coronary lesion morphology on CCTA independent of clinical characteristics and obesity (20). Hwang et al. (22) demonstrated that a greater amount of EAT at baseline CT was an independent predictor of NCP development in asymptomatic individuals. Taken together, these findings demonstrate the utility of measuring EAV to detect high-risk patients.

It remains controversial whether EAV enables the provision of prognostic information in patients with CAD (41, 42). Gitsioudis et al. (41) demonstrated that increased EAV (EAV ≥162.2 cm^3^) is associated with coronary plaque burden and is a predictor of worse outcome independent of risk factors, although the increased EAV did not remain as an independent predictor when coronary artery luminal stenosis was included in the model. Brandt et al. demonstrated that EAT volume showed the improved prediction performance in addition to clinical risk score alone or its combination with CCTA findings (42). Contrastingly, CCTA-derived LAP has been validated by histopathology, demonstrating a close association between LAP and lipid core plaque (43) and outcomes, including mortality and non-fatal myocardial infarction (11, 17). While the detection of obstructive CAD is the central focus of CCTA in patients with symptomatic CAD, the identification of high-risk plaque burden may provide further information on risk stratification beyond luminal stenosis. Romijn et al. showed the importance of adding EAV to CACS in predicting functionally significant stenosis in patients with CAD who underwent CCTA and invasive coronary angiography (23). Our results indicate that the addition of EAV to CACS can be used for risk stratification of patients with a greater plaque burden requiring immediate coronary revascularization and at risk of developing future ACS event.

### Study Limitations

This study has some limitations. First, this study had a small sample size with relatively high Suita scores (48.4 ± 10.8 points). This can be explained by the advanced age of the participants. Moreover, patients had a high prevalence of coronary risk factors, including hypertension (71.5%) and dyslipidemia (73.1%), whereas pharmacological therapeutic interventions were deemed to be insufficient at baseline. Second, to measure the CT attenuation values of coronary plaques, we used a tube voltage of 120 kV in all patients regardless of body weight. To reduce radiation exposure, however, we used the wide-volume scan method with a prospective gating method. Third, the event rate observed in this study was relatively high (4.5%), and this may be explained by the relatively high Suita scores of the participants. Another explanation is that the approximately half of the primary endpoint consisted of a soft endpoint, including unstable angina and coronary revascularization. Fourth, this study did not analyze pericoronary adipose tissue attenuation (PCATA) (39), which has been shown to be an independent predictor of prognosis in patients with CAD. Given that PCATA might be associated with inflammation around coronary arteries, analyzing PCATA will further help in risk stratification beyond the EAT burden. Fifth, although plaque rupture is a major leading cause of ACS and sudden cardiac death, attempts have been made in recent decades to identify patients at risk of ACS caused by plaque erosion, the second leading cause of ACS (8). ACS caused by plaque erosion has been reported to be more common in females. Plaque erosion has fewer inflammatory cells, and is a proteoglycan-rich lesion in contrast to plaque rupture (8). Further *in vivo* investigations are warranted to elucidate the mechanisms and plaque composition in patients with ACS caused by plaque erosion. Novel intracoronary imaging may provide new insights (30, 32). Sixth, cardiac troponin can be elevated even in patients with stable CAD, and has been shown to be associated with increased cardiovascular events (44); however, this study did not include any biomarker analysis that may have provided mechanistic insights regarding the hypothesis of identifying patients at very high risk for developing a primary endpoint carrying both increased LAP volume and elevated CACS and EAT volume. Finally, due to the retrospective nature of the study, the effect of medical therapies on the outcomes was not included in the analysis. A recent CCTA study demonstrated that icosapent ethyl was effective for the reduction of coronary plaque volume assessed using CCTA (45). Accumulating evidence suggests potential therapeutic strategies for reducing EAT (46). These findings suggest that CCTA might be useful for identifying and monitoring patients at a higher risk of future ACS events in response to OMT.

## Conclusions

In conclusion, CCTA-based assessment of EAV, CACS, and LAP could help improve personalized cardiac risk management by administering patient-suited therapy.

## Data Availability Statement

The data that support the findings of this study are available from the corresponding author, KO upon reasonable request.

## Ethics Statement

This study was approved by the ethics committee of Fujiikai Kashibaseiki Hospital. The requirement of obtaining written informed consent from the participants was waived in accordance with the institutional requirements. The study was conducted in accordance with the Declaration of Helsinki.

## Author Contributions

KO and HY conceived this study design and contributed equally to this work. HY performed statistical analysis, interpreted the data, and prepared manuscript draft. KO and HI performed image analysis, interpreted the data, and prepared manuscript draft. KS performed data collection. NK and DF supervised this study and prepared manuscript draft. All authors contributed to the article and approved the submitted version.

## Conflict of Interest

The authors declare that the research was conducted in the absence of any commercial or financial relationships that could be construed as a potential conflict of interest.

## Publisher's Note

All claims expressed in this article are solely those of the authors and do not necessarily represent those of their affiliated organizations, or those of the publisher, the editors and the reviewers. Any product that may be evaluated in this article, or claim that may be made by its manufacturer, is not guaranteed or endorsed by the publisher.
